# Use of bedside echocardiography in the care of critically ill patients - a joint consensus document of the *Associação de Medicina Intensiva Brasileira, Associação Brasileira de Medicina de Emergência*, and *Sociedade Brasileira de Medicina Hospitalar*. Part 1 - Competence in bedside echocardiography

**DOI:** 10.5935/2965-2774.20230307-en

**Published:** 2023

**Authors:** José Augusto Santos Pellegrini, Ciro Leite Mendes, Paulo César Gottardo, Khalil Feitosa, Josiane França John, Ana Cláudia Tonelli de Oliveira, Alexandre Jorge de Andrade Negri, Ana Burigo Grumann, Dalton de Souza Barros, Fátima Elizabeth Fonseca de Oliveira Negri, Gérson Luiz de Macedo, Júlio Leal Bandeira Neves, Márcio da Silveira Rodrigues, Marcio Fernando Spagnól, Marcus Antonio Ferez, Ricardo Ávila Chalhub, Ricardo Luiz Cordioli

**Affiliations:** 1 Department of Intensive Care, Hospital de Clínicas de Porto Alegre, Universidade Federal do Rio Grande do Sul - Porto Alegre (RS), Brazil; 2 Department of Intensive Care, Hospital Universitário Lauro Wanderley - João Pessoa (PB), Brazil; 3 Department of Intensive Care, Hospital Nossa Senhora das Neves - João Pessoa (PB), Brazil; 4 Department of Emergency Medicine, Hospital Geral de Fortaleza - Fortaleza (CE), Brazil; 5 Universidade do Vale do Rio dos Sinos - São Leopoldo (RS), Brazil; 6 Department of Intensive Care, Hospital Nereu Ramos - Florianópolis (SC), Brazil; 7 Cardiovascular Intensive Care Unit, Hospital Cardiopulmonar Instituto D’Or - Salvador (BA), Brazil; 8 Intensive Care Unit, Hospital Universitário de Vassouras - Vassouras (RJ), Brazil; 9 Intensive Care Unit, Hospital Geral Roberto Santos - Salvador (BA), Brazil; 10 Department of Emergency, Hospital de Clínicas de Porto Alegre, Universidade Federal do Rio Grande do Sul - Porto Alegre (RS), Brazil; 11 Department of Hospital Medicine, Hospital Mãe de Deus - Porto Alegre (RS), Brasil; 12 Intensive Care Unit, Hospital Beneficência Portuguesa - Ribeirão Preto (SP), Brazil; 13 Department of Echocardiogram, Hospital Santo Antônio, Obras Sociais Irmã Dulce - Salvador (BA), Brazil; 14 Department of Intensive Care, Hospital Israelita Albert Einstein - São Paulo (SP), Brazil

**Keywords:** Ultrasonography, Critical care, Point-of-care systems, Consensus

## Abstract

The use of echocardiography by physicians who are not echocardiographers has become common throughout the world across highly diverse settings where the care of acutely ill patients is provided. Echocardiographic evaluation performed in a point-of-care manner can provide relevant information regarding the mechanism of causes of shock, for example, increasing the rates of correct diagnosis and allowing for faster informed decision-making than through evaluation methods. Considering that the accurate diagnosis of life-threatening situations is essential for professionals working with acutely ill patients, several international associations recommend that physicians responsible for critically ill patients acquire and develop the ability to perform bedside ultrasound examinations, including echocardiographic examinations. However, there is no consensus in the literature regarding which specific applications should be included in the list of skills for nonechocardiographer physicians. Taking into account the multiplicity of applications of echocardiography in different scenarios related to acutely ill patients; the differences in the published protocols, with regard to both the teaching methodology and competence verification; and the heterogeneity of training among highly diverse specialties responsible for their care at different levels, this consensus document aimed to reflect the position of representatives of related Brazilian medical societies on the subject and may thus serve as a starting point both for standardization among different specialties and for the transmission of knowledge and verification of the corresponding competencies.

## INTRODUCTION

The use of echocardiography by physicians who are not echocardiographers has become common throughout the world across highly diverse settings where the care of acutely ill patients is provided.^(1)^ Echocardiographic evaluation performed in a point-of-care manner can provide relevant information regarding the mechanism of causes of shock, for example, increasing the rates of correct diagnosis and allowing for faster informed decision-making than through other evaluation methods.^(2,3)^

Considering that the accurate diagnosis of life-threatening situations is essential for professionals working with acutely ill patients, several international associations recommend that physicians responsible for critically ill patients acquire and develop the ability to perform bedside ultrasound examinations, including echocardiographic examinations.^(4,5,6,7)^ However, there is no consensus in the literature regarding which specific applications should be included in the list of skills for nonechocardiographer physicians.

Taking into account the multiplicity of applications of echocardiography in different scenarios related to acutely ill patients; differences in the published protocols, with regard to both teaching methodology and competence verification; and the heterogeneity of training among highly diverse specialties responsible for the care of acutely ill patients at different levels, this consensus document aims to reflect the position of representatives of similar Brazilian medical societies on the subject and may thus serve as a starting point both for standardization among different specialties and for the transmission of knowledge and verification of the corresponding skills.

The choice of elaborating a document in consensus format is due to several factors, including the wide use of echocardiography by nonechocardiographers in highly diverse settings in which critically ill patients are cared for; the great variation in regional practice in several aspects;^(8)^ the demand identified by the different medical entities involved for guidance regarding the teaching practices and respective competencies that involve the use of ultrasound by nonechocardiographer physicians, with potential gain in care quality; the scarcity of high-quality evidence to guide the recommendation escalation process; and the lack of a similar position at the national level that represents the Brazilian reality in terms of health system organization, professional training, and equipment availability.^(9)^

The primary focus of this consensus is issues related to the competences in bedside echocardiography by nonechocardiographer physicians. Technical aspects related to the evaluation of left and right ventricular function, diagnosis of shock, and hemodynamic evaluation are addressed in a separate document, complementary to this one.

## METHODS

This document is a collaborative initiative between the *Associação de Medicina Intensiva Brasileira* (AMIB), the *Associação Brasileira de Medicina de Emergência* (ABRAMEDE), and the *Sociedade Brasileira de Medicina Hospitalar* (SOBRAMH). There was no financial support from any source.

The committee initially consisted of representatives from each of these entities and was later structured through the appointment of representatives from each of the entities involved. Each member nominated was required to have recognized experience in the use of ultrasound for cardiovascular evaluation in their daily clinical practice. The previous development of clinical research in this area of knowledge and the practice of teaching ultrasound to medical professionals or students in training were recommended criteria, although not mandatory requirements. The final group consisted of 17 consultants representing the collaborative specialties and from different regions of Brazil.

The questions were selected using the Delphi method.^(10)^ Two of the authors prepared a set of questions that were electronically subjected to three cycles of review by the group. A facilitator assessed the agreement between the individuals and provided individual feedback to each of the consultants about their responses and any questions they might have. Between the second and third consultation cycles, there were no changes in the content of the questions, thus validating them. There were no face-to-face or virtual meetings for this purpose. A set of 28 questions was then created regarding the competences relevant to the use of echocardiography by nonechocardiographer physicians. To follow the consensus process, the modified Delphi method was used.

A systematic review was conducted by two authors independently, with the objective of compiling a theoretical basis for obtaining answers to the chosen questions. Each author gathered original studies on the topics of interest in Portuguese and English. The search results did not include review articles, letters or editorials, or studies in experimental models. The two sets of searches were subjected to a search for duplicates, which were duly excluded. The final product of the search was made available to the committee members. Additional comment on the references of the included articles or individual searches by each consultant was allowed whenever considered necessary by each member of the committee.

The questions were made available to the committee through an electronic form (Google Forms). All questions were prepared using a five-point Likert scale: strongly disagree [1], disagree [2], neutral [3], agree [4], and strongly agree [5]. Consensus was defined *a priori* as the sum of at least 80% of the responses being 1 and 2 or 4 and 5.

The facilitator assessed the coherence of the responses obtained from each member and, in case inconsistencies were identified between the responses that suggested an error in the understanding of the statement or even a mistake in filling out the questionnaire, sent individual responses by e-mail as a form of conference. The questions that did not generate consensus in the first *round* of submissions were forwarded to the committee members for a second round, held 4 weeks after the first round. At the end of each round, all participants received a complete summary of the group voting results for each question evaluated along with their own responses. The individual responses of each member were kept confidential from the other members of the committee at all stages of the process.

The issues that remained without consensus after this stage were subjected to online voting in two virtual meetings, which brought together all the participants of the committee. At this stage, the participants had the opportunity to discuss the particularities of each of the questions and argue about their position. The attributions of the facilitator at this stage were to clarify any doubts of the participants, to allow all participants who wished to have the opportunity to express their views without the need to reach a consensus on any issues, and to compile the results of the votes obtained in each of the steps.

In the virtual meetings, the questions still without consensus in the first two stages were presented to the participants in a grouped manner in two different batches: questions close to consensus (when more than 60% of the answers were 1 and 2 or 4 and 5) and questions Far from consensus (when the responses were distributed such that less than 60% of the responses were 1 and 2 or 4 and 5). The votes were also obtained anonymously through the online platform Mentimeter (http://www.mentimeter.com). After the online voting results, issues that had not yet reached consensus could be put to a new vote only once, provided that the absolute majority of participants agreed.

## RESULTS

All participants answered the questions relevant to each stage, including the virtual meeting, with the exception of the facilitator. Thus, the sum of 16 responses is applied to all questions. In the first round, consensus was reached for 10 of the 28 questions. In the second round, another three questions reached consensus, leaving 15 questions for virtual discussion among the participants. At the end of all steps, there were 17 positive (agreement) and eight negative (disagreement) consensuses; another three questions remained without consensus among the participants (Table 1).

**Table 1:** Consensus issues

Questions	Consensus stage	Strongly disagree	Disagree	Neutral	Agree	Strongly agree
**Competency levels**						
1. The echocardiographic examination performed by a nonspecialist physician has distinct characteristics from the complete examination performed by the echocardiographer	1	0	0	1	3	12
		0%		6.25%	93.75%	
2. The echocardiographic examination performed by a nonspecialist physician can replace a complete examination performed by an echocardiographer	2	8	6	2	0	0
		87.5%		12.5%	0%	
3. Nonspecialist physicians are more agile in obtaining answers compared to a complete examination performed by an echocardiographer	3	1	0	0	1	14
		6.25%		0%	93.75%	
4. A minimum of training is required to perform an echocardiographic evaluation at the bedside	1	0	0	0	0	16
		0%		0%	100%	
5. All medical professionals who work with critically ill patients require training in echocardiography of critically ill patients	1	0	0	1	1	14
		0%		6.2%	93.75%	
6. Different levels of competence should be established for a more appropriate application of training and diagnostic use of echocardiography by nonspecialists	1	0	0	1	1	14
		0%		6.2%	93.75%	
**Basic competence**						
7. The recognition of severe left ventricular dysfunction should be part of the basic competence in bedside echocardiography	1	0	0	0	0	16
		0%		0%	100%	
8. The recognition of mild left ventricular dysfunction should be part of the basic competence in bedside echocardiography	3	16	0	0	0	0
		100%		0%	0%	
9. The quantitative assessment of left ventricular systolic function should be part of the basic competence in bedside echocardiography	3	13	2	1	0	0
		93.75%		6.25%	0%	
10. The evaluation of segmental abnormalities of the left ventricle should be part of the basic competence in bedside echocardiography	3	13	0	2	1	0
		81.25%	12.5%	6.25%		
11. The recognition of right ventricular dysfunction should be part of the basic competence in bedside echocardiography	1	0	1	1	0	14
		6.2%		6.2%	87.5%	
12. The measurement of right chamber pressures should be part of the basic competence in bedside echocardiography	3	12	2	0	2	0
		87.5%		0%	12.5%	
13. The evaluation of the diameter and collapsibility of the inferior vena cava should be part of the basic competence in bedside echocardiography	1	0	0	0	3	13
		0%		0%	100%	
14. Measurement of cardiac output should be part of the basic competence in bedside echocardiography	3	12	1	0	3	0
		81.25%		0%	18.75%	
15. The assessment of diastolic function should be part of the basic competence in bedside echocardiography	3	12	1	0	2	1
		81.25%		0%	13.75%	
16. The recognition of cardiac tamponade should be part of the basic competence in bedside echocardiography	1	0	0	0	1	15
		0%		0%	100%	
17. The use of echocardiography in care during cardiac arrest should be part of the basic competence in bedside echocardiography	1	0	0	1	2	13
		0%		6.2%	93.75%	
18. The assessment of fluid responsiveness should be part of the basic competence in bedside echocardiography	No	2	2	0	3	9
		25%		0%	75%	
19. The recognition of severe valvular heart disease should be part of the basic competence in bedside echocardiography	No	6	0	3	0	8
		37.5%		18.75%	50%	
**Advanced competence**						
20. The recognition of mild left ventricular dysfunction should be part of the advanced competence in bedside echocardiography	2	0	2	1	3	10
		12.5%		6.25%	81.25%	
21. The quantitative assessment of left ventricular systolic function should be part of the advanced competence in bedside echocardiography	2	2	0	1	5	8
		12.5%		6.25%	81.25%	
22. The evaluation of segmental abnormalities of the left ventricle should be part of the advanced competence in bedside echocardiography	1	0	0	2	3	11
		0%		12.5%	87.5%	
23. Cardiac output measurement should only be part of the advanced competence in bedside echocardiography	3	2	0	0	3	11
		12.5%		0%	87.5%	
24. Diastolic function assessment should only be part of the advanced competence in bedside echocardiography	3	2	0	0	2	12
		12.5%		0%	87.5%	
25. The assessment of fluid responsiveness should be part of the advanced competence in bedside echocardiography	3	0	1	2	2	11
		6.25%		12.5%	81.25%	
26. The recognition of severe valvular heart disease should be part of the advanced competence in bedside echocardiography	3	1	2	0	4	9
		18.75%		0%	81.25%	
27. The quantitative evaluation of mild and moderate valvular heart disease should be part of the advanced competence in bedside echocardiography	3	12	2	0	1	1
		87.5%		0%	12.5%	
28. The measurement of right chamber pressures should be part of the advanced competence in bedside echocardiography	No	3	1	0	1	11
		25%		0%	75%	

Questions 1 to 3 refer to conceptual aspects of the echocardiography of critically ill patients in relation to the complete examination performed by the echocardiographer, and the results were as follows:

**1. The echocardiographic examination performed by a nonspecialist physician has distinct characteristics from the complete examination performed by the echocardiographer - 93.75% agreement.**
**2. The echocardiographic examination performed by a nonspecialist physician can replace a complete examination performed by an echocardiographer -87.5% disagreement.**
**3. Nonspecialist physicians are more agile in obtaining answers compared to a complete examination performed by an echocardiographer - 93.75% agreement.**

A complete echocardiographic examination performed by a cardiologist with specific training in echocardiography should be considered the gold standard for the evaluation of cardiac images using ultrasound.^(5,11)^ This test has a wide spectrum of indications and uses multiple technologies, equipment with high capacity for two- and three-dimensional image formation, different types of Doppler, and possibly contrast media.

Bedside echocardiographic evaluation by a nonechocardiographer is intended to be rapid and objective and occur in a specific clinical context, with the objective of answering a specific question among a list of possible diagnoses. It should be used when there is an acute change in the clinical status of the patient.^(5,12)^ In a nonrandomized study, Becker et al. reported higher diagnostic accuracy (an additional 14.8%) with the use of cardiopulmonary ultrasound in the evaluation of patients with shock or respiratory dysfunction in the emergency room; this difference was especially pronounced in patients with a final diagnosis of cardiac origin (94.7 *versus* 40%).^(13)^ Jones et al.^(2)^randomized patients with nontraumatic hypotension admitted to the emergency room to be subjected to an ultrasound protocol immediately or only after initial evaluation. The group where ultrasound was used immediately had fewer of diagnostic hypotheses as the cause of hypotension and a higher proportion of correct diagnoses within 15 minutes of admission. Shokoohi et al.^(14)^ observed that a protocol of ultrasound evaluation of patients with hypotension without a definite diagnosis in the emergency room reduced the diagnostic uncertainty and resulted in a 0.80 agreement with the definitive diagnosis. Zieleskiewicz et al.^(15)^ evaluated the incorporation of portable ultrasound in the evaluation of clinical complications by the Rapid Response Team and observed that the use of ultrasound was associated with a significant increase in the proportion of immediate and adequate diagnoses (94 *versus* 80%) and a shorter implementation time for treatment or conduct deemed necessary; similar results were reported by other authors.^(16,17)^ It is noteworthy that in most protocols studied in this context, echocardiography is performed together with the evaluation of other organs or systems.

The committee participants agreed that there are distinct characteristics between an echocardiographic examination performed by a nonspecialist physician at the bedside and a complete echocardiographic examination performed by an echocardiographer, although the former does not replace the latter. Therefore, a comprehensive approach to critically ill patients should be implemented in an integrative manner, incorporating information obtained through each method.

Questions 4 to 6 specifically address the need for specific training to perform echocardiography in critically ill patients. In all of them, there was a consensus.

**4. A minimum of training is required to perform an echocardiographic evaluation at the bedside - 100% agreement.**
**5. All medical professionals who work with critically ill patients require training in echocardiography of critically ill patients - 93.75% agreement.**
**6. Different levels of competence should be established for a more appropriate application of training and diagnostic use of echocardiography by nonspecialists - 93.75% agreement.**

The performance of bedside echocardiographic exams in critically ill patients should be a skill of physicians of any specialty providing direct care to critically ill patients,^(18,19)^ with the final objective of providing the diagnostic resource at the time the patient needs it. Several international entities support the use of echocardiography as a diagnostic tool by nonechocardiographers.^(4,5,20,21,22)^

In the present document, there was a consensus that a minimum amount of specific training is required so that the physician responsible for the critically ill patient can properly use ultrasound at the bedside for echocardiographic evaluation (100% agreement). Likewise, the participants agreed that it is necessary to define different levels of competence according to the complexity of the measurements or techniques used. Previously, several documents from international associations proposed stratification of competence levels in bedside echocardiography.^(4,23,24)^

Questions 7 to 19 concern basic competence in echocardiography of critically ill patients. In questions 7 to 17, there was consensus (agreement or disagreement), while questions 18 and 19 remained without consensus at the end of the process.

**7. The recognition of severe left ventricular dysfunction should be part of the basic competence in bedside echocardiography - 100% agreement.**
**8. The recognition of mild left ventricular dysfunction should be part of the basic competence in bedside echocardiography - 100% disagreement.**
**9. The quantitative assessment of left ventricular systolic function should be part of the basic competence in bedside echocardiography -93.75% disagreement.**
**10. The evaluation of segmental abnormalities of the left ventricle should be part of the basic competence in bedside echocardiography -81.25% disagreement.**
**11. The recognition of right ventricular dysfunction should be part of the basic competence in bedside echocardiography - 83.75% agreement.**
**12. The measurement of right chamber pressures should be part of the basic competence in bedside echocardiography - 87.5% disagreement.**
**13. The evaluation of the diameter and collapsibility of the inferior vena cava should be part of the basic competence in bedside echocardiography -100% agreement.**
**14. Measurement of cardiac output should be part of the basic competence in bedside echocardiography - 81.25% disagreement.**
**15. The assessment of diastolic function should be part of the basic competence in bedside echocardiography - 81.25% disagreement.**
**16. The recognition of cardiac tamponade should be part of the basic competence in bedside echocardiography - 100% agreement.**
**17. The use of echocardiography in care during cardiac arrest should be part of the basic competence in bedside echocardiography - 93.75% agreement.**

Basic-level echocardiographic evaluation aims to answer a limited number of clinical questions commonly encountered by physicians who work with critically ill patients. The evaluation is directed to the clinical context of the patient and should be repeated after specific therapeutic interventions.^(4,25)^ Studies that evaluated training curricula in the ultrasonography of critically ill patients performed better and were more reproducible when they comprised a smaller number of items and performed the study qualitatively.^(26,27,28,29)^ There was a consensus that the recognition of severe left ventricular (LV) dysfunction and right ventricular (RV) dysfunction and the evaluation of the diameter and collapsibility of the inferior vena cava should be part of the basic competence. Likewise, the recognition of cardiac tamponade and the use of echocardiography during care for cardiac arrest should be skills included in the basic competencies.

In contrast, there was disagreement that the recognition of mild LV dysfunction (or even its quantitative assessment), the assessment of diastolic function, the measurement of right chamber pressures and cardiac output, or the assessment of LV segmental function should be part of the list of competencies. Many of these applications of bedside echocardiography have in common the use of quantitative tools and knowledge of the particularities related to the use of Doppler imaging. In turn, the correlation between a test performed by a nonspecialist and an echocardiographer is low or moderate for the assessment of LV segmental function.^(29)^

**18. The assessment of fluid responsiveness should be part of the basic competence in bedside echocardiography - no consensus.**
**19. The recognition of severe valvular heart disease should be part of the basic competence in bedside echocardiography - no consensus.**

The assessment of fluid responsiveness has become a fundamental part of the care of critically ill patients.^(30)^

The careful identification of those patients most likely to show increased cardiac output in response to the administration of a given aliquot of fluid is in line with the objective of minimizing indiscriminate water overload in nonresponders, which is associated with worse outcomes.^(31)^

Several maneuvers have been used to identify fluid-responsive patients, using methods that simulate a water challenge (passive leg elevation, e.g., “minibolus”) or explore the behavior of the heart-lung interaction (e.g., end-expiratory occlusion, peak aortic flow). To properly perform these maneuvers, as a rule, it is necessary to use cardiac output monitoring in real time, for which bedside echocardiography is one of the main tools.

However, this application of echocardiography requires a series of knowledge of heart-lung interactions and the use of specific requirements for the applicability of each maneuver. Furthermore, obtaining quantitative measurements at different times of the respiratory cycle or in response to different positions or maneuvers requires the examiner to be able to quickly and accurately obtain images at the right time. These are possible reasons for the lack of consensus. However, given the representativeness of this evaluation in the care of critically ill patients, even with the limitations described and the absence of consensus, the committee participants understand that fundamental concepts of fluid responsiveness evaluation should be part of the physician’s skills at the level of basic competence.

Although the identification of severe valvular heart disease is frequent in the general population, especially in the elderly, and sufficiently relevant for the proper management of critically ill patients, few studies have evaluated the accuracy of bedside echocardiogram for the identification of valvular heart disease, with conflicting results.^(7,32,33)^

However, detailed and quantitative evaluation requires mastery of tools such as continuous Doppler imaging and specific methods for grading valvular lesions. The correlation between an examination by a nonspecialist and an echocardiographer for valvular heart disease evaluation was reported as low to moderate in a recent systematic review.^(29)^ Thus, the in-depth evaluation of the functional evaluation of valvular heart disease should be considered the scope of the echocardiographer.

Items 20 to 28 address aspects related to advanced competence. Question 28 was the only question of this block to remain without consensus at the end of all stages of the process.

**20. The recognition of mild left ventricular dysfunction should be part of the advanced competence in bedside echocardiography -81.25% agreement.**
**21. The quantitative assessment of left ventricular systolic function should be part of the advanced competence in bedside echocardiography -81.25% agreement.**
**22. The evaluation of segmental abnormalities of the left ventricle should be part of the advanced competence in bedside echocardiography -87.5% agreement.**
**23. Cardiac output measurement should only be part of the advanced competence in bedside echocardiography - 87.5% agreement.**
**24. Diastolic function assessment should only be part of the advanced competence in bedside echocardiography - 87.5% agreement.**
**25. The assessment of fluid responsiveness should be part of the advanced competence in bedside echocardiography - 81.25% agreement.**
**26. The recognition of severe valvular heart disease should be part of the advanced competence in bedside echocardiography - 81.25% agreement.**
**27. The quantitative evaluation of mild and moderate valvular heart disease should be part of the advanced competence in bedside echocardiography - 87.25% disagreement.**

Advanced-level echocardiographic evaluation proposes a more comprehensive hemodynamic evaluation and more precise guidance and treatment of critically ill patients.^(1,4,25)^

The advanced level presupposes a mastery of the different techniques of transthoracic echocardiography, including different Doppler tools, and may also include transesophageal echocardiography in areas with greater equipment availability.^(4)^ There was consensus (with agreement) in seven of the nine questions evaluating the advanced competencies.

There was negative consensus (disagreement) regarding the assessment of mild to moderate valvular heart disease. The evaluation of these conditions does not fall within the scope of nonechocardiographers and should therefore be reserved for elective and complete examination.

**28. Measurement of right chamber pressures should be part of the advanced competence in bedside echocardiography - no consensus.**

There was no consensus regarding the incorporation of right chamber pressure measurement as part of advanced-level skills. Although they may be useful for the evaluation of hemodynamically unstable patients and those with the potential to develop pulmonary hypertension, the estimation of right atrial pressure by evaluating the dynamics of the inferior vena cava (essential for obtaining the other related pressure parameters) suffers from a number of problems and limitations in critically ill patients, from an inadequate window and positioning to reduced reliability of the method when the patient is ventilated with positive pressure. The alternative for these patients remains invasive monitoring through catheters inserted into the right atrium or through a pulmonary artery catheter.

Due to the relevance of this evaluation in severely hypoxemic patients or patients with compromised ventilatory mechanics, in addition to the borderline result obtained, the participants of the committee understand that the measurement of right chamber pressures should be part of the skills of the physician at the advanced level of competence in echocardiography of critically ill patients.

## CONCLUSION

The purpose of this project was to synthesize information and discuss points of interest that may improve the development of bedside echocardiography by physicians who are not specialists in echocardiography. The issues addressed throughout the text may reflect uncertainties and be influenced by personal points of view; however, the rigorous methodology for obtaining consensus aims to mitigate personal issues and identify the position of a group of people dedicated to the development of bedside echocardiography.

It is essential to emphasize that consensus documents are based on the opinions of experts and are primarily informative and educational. Consensus documents are not guidelines and have the ultimate goal of creating opportunities for improvement in the quality of care in their associated topic.

Using the Delphi method, participants from medical associations representing different areas of expertise responsible for the care of critically ill patients reached consensus on most questions pertinent to the competencies related to the use of bedside echocardiography by physicians who are not specialists in echocardiography. This document can serve as a tool to guide the transmission of knowledge on the subject and the development of skills relevant to each of the levels of competence.
